# Cross-sectional associations between questionnaire-measured physical activity and tissue doppler indices of left ventricular diastolic function

**DOI:** 10.1186/s12872-023-03559-3

**Published:** 2023-10-27

**Authors:** Lina Su, Xiaodi Yan, Yongmei Pan, Songna Yang

**Affiliations:** Department of Cardiology, Peking University People’s Hospital, 11 Xizhimen South St, Beijing, 100044 PR China

**Keywords:** Physical activity, Left ventricular diastolic dysfunction, Tissue doppler indices

## Abstract

**Background:**

The prevalence of left ventricular (LV) diastolic dysfunction has been increasing over the past decade, and to date, effective pharmacotherapies that enhance LV diastolic function have not yet been identified. Though some data has demonstrated the beneficial effects of exercise training on LV diastolic function, little is known about the adaptations of diastolic function to daily physical activity (PA). Accordingly, our study aimed to investigate the impact of daily PA on tissue Doppler indices of LV diastolic function.

**Methods:**

A total of 432 participants were enrolled for clinically indicated echocardiography from July 2019 to July 2020 at Peking University People’s Hospital. Participants aged ≥ 18 years were included if they had stable PA in the past six months and normal LV systolic function. A questionnaire was used to collect demographic characteristics, medical history, and daily PA. According to PA Guidelines for Americans, we identified these participants into low-intensity PA (LPA) group and moderate-high-intensity PA (MHPA) group. Propensity score matching (PSM) was performed to match potential confounding factors between the two groups. The clinical characteristics and echocardiographic parameters between LPA group and MHPA group were compared using student’s t-test, Mann-Whitney U test, and chi-square test as appropriate.

**Results:**

After matching potential confounding factors using PSM with a 1:3 matching ratio, our final analysis included 86 cases in the MHPA group and 214 cases in the LPA group. All demographic characteristics and comorbidities were statistically similar between the two groups. Compared to the LPA group, the MHPA group showed higher septal e’ (7.9 ± 2.9 cm/s versus 7.2 ± 2.6 cm/s, *P* = 0.047). Other echocardiographic parameters associated with LV diastolic function concerning lateral e’ and average E/e’, also trended towards improved LV diastolic function in the MHPA group, but failed to reach statistical significance.

**Conclusions:**

Our study demonstrated that moderate-high-intensity daily PA was associated with improved septal e’, suggesting that moderate-high-intensity PA could potentially ameliorate LV diastolic dysfunction.

**Supplementary Information:**

The online version contains supplementary material available at 10.1186/s12872-023-03559-3.

## Introduction

The prevalence of left ventricular (LV) diastolic dysfunction (DD) is increasing relative to the aging population with greater cardiometabolic comorbidities in recent years [1]. Current knowledge demonstrates that DD is strongly associated with impaired exercise capacity and reduced quality of life [2], ultimately resulting in the development of heart failure with preserved ejection fraction (HFpEF) and even adverse outcomes in healthy individuals [3]. However, effective pharmacotherapies have not yet been identified to enhance LV diastolic function [4, 5]. Though some data has demonstrated the beneficial effects of exercising training on LV diastolic function [6], [7–9], little is known about the adaptations of diastolic function to daily physical activity (PA). Echocardiography is the primary tool to identify and classify abnormal diastolic function in clinical practice [10]. Compared with traditional Doppler values, tissue Doppler indices of LV diastolic function have been proven to be more accurate, sensitive, and less load-dependent [11]. The purpose of this study was therefore to investigate the influences of comprehensive daily PA, encompassing leisure time, occupation, transportation, and household domains, on tissue Doppler indices of LV diastolic function.

## Methods

### Study population

From July 2019 to July 2020, 432 participants aged ≥ 18 years with stable PA in the past six months were recruited for clinically indicated echocardiography at Peking University People’s Hospital. We excluded individuals with conditions such as atrial fibrillation/flutter, cardiomyopathy, severe valvular disease, heart failure with reduced ejection fraction, myocardial infarction, pulmonary hypertension, or severe anemia. Additionally, those who either had incomplete echocardiographic data or declined to participate in the questionnaire were also excluded. Prior to their involvement, we obtained oral informed consent from all subjects.

### Data collection

A questionnaire was used for collecting information concerning age, sex, height, body weight, self-reported use of alcohol, smoking, medical history, and daily PA. Body mass index (BMI) was calculated as weight/height^2^ (kg/m^2^). Body surface area (BSA) was computed as 0.0061×height(m) + 0.0128×weight(kg)-0.1529. According to American Heart Association’s recommendations for assessing PA [12], [13], we took the international PA questionnaire (IPAQ) to evaluate recent PA, while the Compendium of PA was utilized to measure lifetime PA. The IPAQ is a globally recognized, standardized, and culturally adaptable tool used across various populations [14]. Participants completed the comprehensive self-report version of the questionnaire, which covers PA related to leisure time, occupation, transportation, household activities, as well as sedentary time. Despite the IPAQ being originally designed for individuals aged 15 to 69, its reliability in assessing PA among older adults has been adequately established [15]. Conforming to the IPAQ scoring protocol, different intensity activity levels were calculated as the metabolic equivalent (MET) and then weighted by reported minutes in every category, thereby quantifying PA expressed as MET-minutes per week. In accordance with the 2018 PA Guidelines for Americans, at least 75 min of vigorous-intensity or 150 min of moderate-intensity aerobic activities (equivalent to 600 MET-minutes) per week are recommended for significant health benefits [12, 16]. Based on these recommendations, participants who met these criteria were distributed to the moderate-high-intensity PA (MHPA) group, while those who didn’t were placed in the low-intensity PA (LPA) group. For lifetime PA, we followed a similar quantification process but weighted each category by reported hours instead of minutes, and finally normalized by age to account for accumulated activity over time.

### Echocardiography

Echocardiography was performed and analyzed by two highly experienced and qualified sonographers using a GE Vivid E9 machine (GE Medical, Horten, Norway) with an M5S transducer. All images and analyses were reviewed by an independent cardiologist who was not aware of the patient’s clinical information and PA. In case of a discrepancy, the images were reviewed by another cardiologist. All echocardiography measurements were conducted and analyzed in accordance with the 2016 American Society of Echocardiography (ASE) recommendations [17]. M-mode measures of LV internal diameter, wall thickness of interventricular septum and LV posterior wall, and left atrial (LA) anteroposterior diameter were all collected from parasternal long-axial view. LV ejection fraction (LVEF) was computed according to the formula: (LV end-diastolic volume [LVEDV] - LV end-systolic volume)/LVEDV. LV mass (LVM) was calculated with the formula of Devereux et al, and LVM index (LVMI) was calculated as follows: LVMI = LVM/BSA [14]. The early (E) and late (A) mitral inflow velocities were both obtained with pulsed-wave Doppler in the apical four-chamber view while the peak velocity of tricuspid regurgitation (TR) was recorded in the parasternal short-axial view. Tissue Doppler imaging was performed to measure the peak early diastolic tissue velocities of the septal (septal e’) and lateral mitral annulus (lateral e’) from the apical four-chamber view.

### Statistical analysis

Propensity score matching (PSM) was estimated using non-parsimonious multiple logistic regression model. To balance potential confounding factors between LPA group and MHPA group, PSM was adopted across key variables including age, gender, BMI, coronary heart disease, hypertension, diabetes mellitus, hypercholesterolemia, renal disease, and smoking history. These variables were chosen for matching due to their potential association with DD. In our propensity analysis, MHPA cases were matched in a 1:3 ratio to LPA controls with a standard caliper width of 0.2.

All continuous variables were presented as mean ± standard or as median and interquartile range for variables with skewed distribution. Categorical variables were expressed as numbers and percentages. The clinical characteristics and echocardiographic parameters between LPA group and MHPA group in matched or unmatched cohorts were compared using student’s t-test for continuous variables with normal distribution or Mann-Whitney U test for continuous variables with skewed distribution, and chi-square test for categorical variables. The correlation analysis with Spearman’s correlation coefficient was used to determine the relationships between septal e’ and various PA among the matched cohorts. All statistical analyses were performed with SPSS statistic version 23.0 (IBM Co., Chicago, IL, USA). Two-sided *P* values < 0.05 were considered statistically significant.

## Results

A total of 432 patients were included in this study. According to PA status, we finally identified 338 participants in the LPA group and 86 participants in the MHPA group. Among the unmatched cohort (Table 1), the MHPA group demonstrated a higher E/A ratio (1.2 ± 0.5 versus 1.0 ± 0.4; *P* = 0.011), septal e’ (7.9 ± 2.9 versus 6.9 ± 2.4 cm/s; *P* = 0.002) and lateral e’ (10.4 ± 3.5 versus 9.4 ± 3.1 cm/s; *P* = 0.009), but a lower E/e’ ratio (9.1 ± 3.1 versus 10.0 ± 2.9; *P* = 0.018) compared to the LPA group. Although these discrepancies between the two groups seemed modest, they all reached statistical significance, consistently indicating better LV diastolic function in the MHPA group. However, no significant differences were found in LA, TR, LV internal diameter during diastole (LVIDd), interventricular septum dimension during diastole (IVSd), LV posterior wall thickness during diastole (LVPWd), LVEF, and LVMI between the two groups. It was worth noting that the LPA group was significantly older and had a greater percentage of female participants compared to the MHPA group. Besides, hyperlipidemia was more common in the LPA group. These factors have been currently known as potential risk factors for DD. Other demographic data were largely consistent between the two groups.

**Table 1:** Demographic and clinical characteristics in unmatched cohorts

Variable	LPA (n = 346)	MHPA(n = 86)	*P* value
Male sex	140 (41%)	46 (54%)	0.044
Age (years)	54 ± 14	50 ± 14	0.006
Body mass index (kg·m^2^)	23.7 ± 4.4	23.2 ± 3.4	0.276
Body surface are (m^2^)	1.69 ± 0.24	1.71 ± 0.19	0.505
**Medical history**			
Hypertension	123 (36.4%)	27 (31.4%)	0.387
Hypercholesterolemia	93 (27.5%)	8 (9.3%)	< 0.001
Diabetic mellitus	82 (24.3%)	18 (20.9%)	0.516
Coronary heart disease	18 (5.3%)	3 (3.5%)	0.483
Renal disease	4 (1.2%)	0 (0%)	0.587
Rheumatic disease	29 (8.6%)	3 (3.5%)	0.111
Lung disease	4 (1.2%)	0 (0%)	0.587
Tumor disease	10 (3.0%)	2 (2.3%)	1.000
Alcoholism history	23 (6.8%)	11 (12.8%)	0.068
Smoking history	111 (32.8%)	31 (36.0%)	0.574
**Echocardiogram**			
Left atrial diameter (cm)	3.40 ± 0.48	3.40 ± 0.46	0.999
LVIDd (cm)	4.6 ± 0.5	4.7 ± 0.6	0.158
IVSd (cm)	0.83 ± 0.12	0.82 ± 0.15	0.626
LVPWd (cm)	0.79 ± 0.11	0.80 ± 0.12	0.467
LVEF (%)	71.0 ± 5.9	70.0 ± 6.5	0.275
LVMI (g/m^2^)	73.3± 24.8	74.1 ± 20.0	0.773
Peak E wave (cm/s)	74.0 ± 17.7	75.8 ± 18.8	0.409
Peak A wave (cm/s)	76.8 ± 19.5	70.4 ± 17.7	0.006
E/A	1.0 ± 0.4	1.2 ± 0.5	0.011
Peak e’ septal (cm/s)	6.9 ± 2.4	7.9 ± 2.9	0.002
Peak e’ lateral (cm/s)	9.4 ± 3.1	10.4 ± 3.5	0.009
Peak E/e’ average	10.0 ± 2.9	9.1 ± 3.1	0.018
TR (m/s)	2.1 ± 0.4	2.0 ± 0.4	0.107

Values are presented as mean ± SD, or n (%). LIPA = low-intensity physical activity, MHPA = moderate-high-intensity physical activity, LVIDd = left ventricular internal diameter during diastole, IVSd = interventricular septum dimension during diastole, LVPWd = left ventricular posterior wall thickness during diastole, LVEF = LV ejection fraction, LVMI = left ventricular mass index, TR = peak velocity of tricuspid regurgitation

Given the discrepant risk factors between the two groups, PSM was further performed and 1:3 balanced cohorts were generated with 86 cases in the MHPA group and 214 cases in the LPA group. The matching was based on specific variables detailed in the Methods section. Post-matching, all demographic characteristics and comorbidities were statistically comparable between the two groups (Table 2). Within the matched cohorts, the MHPA group exhibited a higher septal e’ (7.9 ± 2.9 cm/s versus 7.2 ± 2.6 cm/s, *P* = 0.047) than the LPA group. Other echocardiographic parameters, such as lateral e’ and average E/e’ related to LV diastolic function, demonstrated a tendency towards improved LV diastolic function in the MHPA group, though they did not achieve statistical significance.

**Table 2:** Demographic and clinical characteristics in propensity score-matched cohorts

Variable	LPA (n = 214)	MHPA (n = 86)	*P* value
Male sex	105 (49%)	46 (53%)	0.488
Age (years)	52 ± 15	50 ± 14	0.110
Body mass index (kg·m^2^)	23.4 ± 4.3	23.2 ± 3.4	0.728
Body surface are (m^2^)	1.69 ± 0.25	1.70 ± 0.19	0.609
**Medical history**			
Hypertension	76 (35.5%)	27 (31.4%)	0.497
Hypercholesterolemia	23 (10.7%)	8 (9.3%)	0.710
Diabetic mellitus	52 (24.3%)	18 (20.9%)	0.533
Coronary heart disease	9 (4.2%)	3 (3.5%)	0.774
Renal disease	0	0	NA
Rheumatic disease	9 (4.2%)	3 (3.5%)	0.774
Lung disease	4 (1.2%)	0 (0%)	0.202
Tumor disease	5 (2.3%)	2 (2.3%)	0.996
Alcoholism history	15 (7.0%)	11 (12.8%)	0.108
Smoking history	74 (34.6%)	31 (36.0%)	0.810
**Echocardiogram**			
Left atrial diameter (cm)	3.37 ± 0.50	3.40 ± 0.46	0.665
LVIDd (cm)	4.6 ± 0.5	4.7 ± 0.6	0.377
IVSd (cm)	0.82 ± 0.12	0.82 ± 0.15	0.920
LVPWd (cm)	0.78 ± 0.11	0.80 ± 0.12	0.261
LVEF (%)	71.0 ± 6.2	70.0 ± 6.5	0.290
LVMI (g/m^2^)	73.2 ± 24.2	74.1 ± 20.0	0.251
Peak E wave (cm/s)	74.2 ± 17.9	75.8 ± 18.8	0.480
Peak A wave (cm/s)	74.7 ± 19.4	70.4 ± 17.7	0.078
E/A	1.1 ± 0.4	1.2 ± 0.5	0.108
Peak e’ septal (cm/s)	7.2 ± 2.6	7.9 ± 2.9	0.047
Peak e’ lateral (cm/s)	9.7 ± 3.3	10.4 ± 3.5	0.069
Peak E/e’ average	8.3 ± 2.8	7.8 ± 3.1	0.121
TR (m/s)	2.1 ± 0.4	2.0 ± 0.4	0.107

Values are presented as mean ± SD, or n (%). NA = not available, LIPA = low-intensity physical activity, MHPA = moderate-high-intensity physical activity, LVIDd = left ventricular internal diameter during diastole, IVSd = interventricular septum dimension during diastole, LVPWd = left ventricular posterior wall thickness during diastole, LVEF = LV ejection fraction, LVMI = left ventricular mass index, TR = peak velocity of tricuspid regurgitation

Notably, the MHPA group engaged in significantly more moderate-high-intensity and lifetime PA, coupled with less low-intensity PA and fewer sedentary periods compared to the LPA group (Table 3). Moreover, the MHPA group reported significantly more PA in working, transportation, and leisure domains relative to the LPA group (Table 3). The differences in PA between the two groups after matching mirrored the results obtained before (Table S1). We further executed a Spearman correlation analysis to examine the relationship between various types of PA and septal e’ within the matched cohorts. Our findings indicated that enhanced septal e’ was significantly correlated with higher levels of moderate-high-intensity and lifetime PA, but inversely related to low-intensity PA and sedentary time (Table 4). Interestingly, moderate-high-intensity PA exhibited a more profound correlation with septal e’ (r = 0.185, *P* = 0.001) compared to lifetime PA (r = 0.132, *P* = 0.022).

**Table 3:** Physical activities and sedentary times in propensity score-matched cohorts

Variable ((MET-minutes/week)	LPA (n = 214)	MHPA (n = 86)	*P* value
Work domain	0 [0,0]	0 [0,3360]	< 0.001
Transportation domain	0 [0,0]	0 [0,852]	< 0.001
Leisure domain	480 [0,1386]	840 [0,1710]	0.006
Domestic domain	630 [0,2520]	630 [0,2520]	0.911
Low-intensity physical activity	1458 [630,3150]	1260 [0,2551]	0.059
Moderate-high-intensity physical activity	0 [0,0]	1800 [960,4200]	< 0.001
Total recent physical activity	1536 [660,3150]	3777 [2164,7425]	< 0.001
Total siting time (minutes/week)	5876 ± 1065	5245 ± 1255	< 0.001
Lifetime physical activity (MET-hours/week/year)	1.63 [0.60,4.11]	3.15 [1.33,7.92]	< 0.001

Values are presented as mean ± SD or median (IQR). LPA = low-intensity physical activity, MHPA = moderate-high-intensity physical activity, MET = metabolic equivalent

**Table 4:** Spearman correlation analysis of physical activity variables with septal e’ relation in propensity score-matched cohorts (n = 300)

Variables	r	*P* value
Low-intensity physical activities	-0.178	0.002
Moderate-high-intensity physical activities	0.185	0.001
Total siting time	-0.050	0.384
Lifetime physical activity	0.132	0.022

## Discussion

The current study sought to evaluate the effects of daily PA on tissue Doppler indices of LV diastolic function in a cross-sectional population. Our primary finding indicated that moderate-high-intensity PA correlated with improved septal e’, suggestive of enhanced LV diastolic function.

Although several exercise studies have reported on diastolic indices, the impact of exercise or PA on diastolic function remains controversial. The Ex-DHF trial revealed that exercise training significantly improves diastolic E/e’ index in HFpEF patients, which is the first randomized multicenter trial to determine the benefits of exercise training on LV diastolic function [18]. However, a recent meta-analysis of randomized controlled trials in HFpEF patients reported that exercise training enhances exercise capacity without improvement in diastolic function [19]. In our study, we also did not identify a significant change in E/e’. Nonetheless, we discovered that moderate-high-intensity PA was associated with an improved diastolic septal e’ index. This result aligns with a previous work examining LV diastolic function in response to lifelong exercise [20]. The aforementioned divergent results might be attributed to several factors, including variations in the intensity, duration, and types of exercise training or PA, different observed indices of LV diastolic function, as well as the natural heterogeneity of abnormal diastolic function.

DD typically results from impaired LV relaxation and increased LV chamber stiffness. Furthermore, elevated LV filling pressure is the strongest evidence in favor of well-developed DD [21]. For this reason, cardiac catheterization to measure LV filling pressures has been perceived as the gold standard for the assessment of DD [19]. The term “LV filling pressures” is often synonymous with pulmonary capillary wedge pressure (PCWP) [10]. One previous study explored the correlation between echocardiographic indices and PCWP in healthy volunteers, concluding that septal e’ shows a stronger correlation with PCWP (r = 0.81, *P* < 0.001) than either lateral e’ (r = 0.63, *P* < 0.05) or E/e’(r = 0.14, *P* > 0.05) [22]. Another study showed that septal e’ provides more consistently obtainable and less variable measurements than lateral e’ [23]. Collectively, these findings suggest that septal e’ may be a more reliable index of diastolic function. Likewise, in patients post-myocardial infarction, Ricardo et al proved that septal e’ has the strongest echocardiographic relation to exercise capacity, assessed via oxygen consumption (r = 0.42, *P* < 0.001) [24]. These findings might help explain why, in our cohort of subjects, moderate-high-intensity PA was associated only with improved septal e’, but not with other diastolic function indices. Nevertheless, the underlying pathophysiologic mechanism explaining the close association between septal e’ and diastolic function remains unknown. While we can conclude from our data that PA is associated with an improved diastolic septal e’ index suggesting improved diastolic function, it would be inaccurate to say that this is conclusive evidence of improved diastolic function as no one single non-invasive index hitherto is a perfect marker of diastolic function.

Besides, left atrial volume index (LAVI) has been recognized as one of comprehensive measurements of diastolic function. However, our study primarily investigated the association between PA and LV diastolic function, rather than the correlation between left atrium size and LV diastolic function. While exercise can enhance LV diastolic function, it also contributes to an enlarged left atrium [25]— a paradoxical situation since such enlargement typically indicates DD. Due to this complexity, we did not include LAVI in our study to avoid potential confusion in interpreting its changes.

In this study, we also demonstrated a modest association between moderate-high-intensity PA and septal e’ (r = 0.185, *P* = 0.001). Conversely, both low-intensity PA and sedentary time were inversely related, thereby substantiating that a sedentary lifestyle contributes to cardiovascular disease. Given that diastolic function is affected by numerous factors such as age, gender, hypertension, diabetes, and PA [26, 27], it seems reasonable to attribute the moderate correlation coefficients between PA and the diastolic septal e’ index to these varying factors.

Most trials have focused on the effectiveness of center-based supervised exercise on LV diastolic function [19, 28]. Few home-based exercise trials exist, and those that do mainly focus on intensive exercise or recreational activities rather than comprehensive PA [29]. The low adherence rates to these existing exercise protocols have raised significant concerns in long-term exercise intervention studies [20]. Indeed, PA is primarily derived from four common domains concerning occupational, domestic, transportation, and leisure time [12]. It is obvious that an increase in PA in one domain cloud be offset by a decreased activity in another. By integrating PA into various domains according to an individual’s lifestyle, we can significantly boost adherence to exercise protocols. In our study, the MHPA group had markedly more PA in work, transportation, and leisure domains compared to the LPA group. Therefore, emphasis should not solely be placed on leisure PA; the levels of PA in other domains also warrant consideration given their potential contributions to improved diastolic function.

To our knowledge, this study is the first to investigate the impacts of comprehensive daily PA on diastolic function. In this research, PA was quantified by calculating the amount of different intensity activity levels in four domains, measured in MET, and weighted by the reported minutes of PA conducted each week. We observed that 75 minutes of vigorous-intensity or 150 minutes of moderate-intensity PA (equivalent to 600 MET-minutes) per week is associated with an improved diastolic septal e’ index. This novel finding supports and strengthens the assertions of the 2018 PA Guidelines for Americans. Importantly, it was noted that the MHPA group engaged in more lifetime PA and had less sedentary time, factors that may partly contribute to improved diastolic function [30].

Despite these findings, the literature thus far cannot elucidate the mechanisms by which PA improves LV diastolic function. Owing to the known beneficial effects on reducing peripheral vascular resistance and arterial stiffening, it is hypothesized that PA could enhance LV diastolic function by decreasing LV afterload and diastolic filling pressure [31]. Further evidence suggested that PA may play a cardioprotective role in preventing DD by reversing endothelial dysfunction, oxidative stress, and insulin resistance, thereby restoring mitochondrial abnormality and reducing cardiac fibrosis [32–34]. Nonetheless, establishing a direct cause-and-effect relationship between PA and DD necessitates more extensive mechanistic studies.

Several potential limitations of this study should be acknowledged. Firstly, the relatively small sample size was a significant limitation and could have restricted our ability to detect meaningful group differences. The evaluation of LV diastolic function via echocardiography relies on multiple indices, and the solitary enhanced septal e’ in the MHPA group was not sufficient to affirm the effectiveness of PA on LV diastolic function. Thus, larger-scale trials are necessary to clarify the role of PA in enhancing LV diastolic function. Secondly, self-reported PA gathered from questionnaires may be subject to recall and social desirability biases. As electronic devices advance, device-measured PA will provide more accurate and valid data for assessing PA. Thirdly, the cross-sectional study design precludes assessment of longitudinal changes in diastolic function adaptations to PA, and we cannot establish the direction of the association or causation. Lastly, though we applied PSM to adjust covariates, the possibility of residual confounding cannot be entirely excluded.

## Conclusions

The present study revealed that moderate-high-intensity PA, originating from occupational, leisure, and transportation domains, correlated positively with enhanced septal e’. This implied that engaging in moderate-high-intensity PA could potentially ameliorate LV diastolic function. For a more comprehensive understanding, larger-scale studies utilizing objective assessment methods for PA are needed to be further studied in the future.

### Electronic supplementary material

Below is the link to the electronic supplementary material.

Supplementary Material 1

## Data Availability

The datasets generated and analyzed during the current study are available from the corresponding author upon reasonable request.

## Abbreviations

LV: Left ventricular
DD: Diastolic dysfunction
HFpEF: Heart failure with preserved ejection fraction
PA: Physical activity
BMI: Body mass index
BSA: Body surface area
IPAQ: International physical activity questionnaire
MET: Metabolic equivalent
MHPA: Moderate-high-intensity physical activity
LPA: Low-intensity physical activity
ASE: American Society of Echocardiography
LVEF: Left ventricular ejection fraction
LVEDV: Left ventricular end-diastolic volume
LVM: Left ventricular mass
LVMI: Left ventricular mass index
TR: Tricuspid regurgitation
PSM: Propensity score matching
LVIDd: Left ventricular internal diameter during diastole
IVSd: Left ventricular posterior wall thickness during diastole
LVPWd: Left ventricular posterior wall thickness during diastole
PCWP: Pulmonary capillary wedge pressure
LAVI: Left atrial volume index
