# Supplementary material for: Cross-sectional associations between questionnaire-measured physical activity and tissue doppler indices of left ventricular diastolic function
Source: BMC Cardiovasc Disord. 2023 Oct 27;23:527. doi: 10.1186/s12872-023-03559-3 (PMC10612190; doi:10.1186/s12872-023-03559-3)
Supplement: Supplementary file 1 — Supplementary Material 1 [file 12872_2023_3559_MOESM1_ESM.docx]

**Table S1** Physical activities and sedentary times in unmatched cohorts

| Variable ((MET-minutes/week) | LPA (n=346) | MHPA (n=86) | *P* value |
| --- | --- | --- | --- |
| Work domain | 0 [0,0] | 0 [0,3360] | <0.001 |
| Transportation domain | 0 [0,0] | 0 [0,852] | <0.001 |
| Leisure domain | 462 [0,1206] | 840 [0,1710] | 0.002 |
| Domestic domain | 735 [0,2520] | 630 [0,2520] | 0.604 |
| Low intensity | 1656 [630,3444] | 1260 [0,2551] | 0.015 |
| Moderate-high intensity | 0 [0,0] | 1800 [960,4200] | <0.001 |
| Total recent physical activity | 1638 [630,3365] | 3777 [2164,7425] | <0.001 |
| Total siting time (minutes/week) | 5825 ± 1060 | 5245 ± 1255 | 0.023 |
| Lifetime physical activity  (MET-hours/week/year) | 1.70 [0.67,4.43] | 3.15 [1.33,7.92] | 0.004 |

Values are presented as mean ± SD or median (IQR). LPA = low-intensity physical activity, MHPA = moderate-high-intensity physical activity, MET = metabolic equivalent
